# Identifying latent activity behaviors and lifestyles using mobility data to describe urban dynamics

**DOI:** 10.1140/epjds/s13688-023-00390-w

**Published:** 2023-05-18

**Authors:** Yanni Yang, Alex Pentland, Esteban Moro

**Affiliations:** 1 Department of Computing, The Hong Kong Polytechnic University, Hong Kong, China; 2 Connection Science, Institute for Data Science and Society, Massachusetts Institute of Technology, Cambridge, MA United States; 3 Grupo Interdisciplinar de Sistemas Complejos (GISC), Department of Mathematics, Universidad Carlos III de Madrid, Leganés, Madrid, Spain

**Keywords:** Mobility data, Lifestyles, Topic analysis, Non-negative matrix factorization, Segregation, Health Risk, Transportation, Census

## Abstract

Urbanization and its problems require an in-depth and comprehensive understanding of urban dynamics, especially the complex and diversified lifestyles in modern cities. Digitally acquired data can accurately capture complex human activity, but it lacks the interpretability of demographic data. In this paper, we study a privacy-enhanced dataset of the mobility visitation patterns of 1.2 million people to 1.1 million places in 11 metro areas in the U.S. to detect the latent mobility behaviors and lifestyles in the largest American cities. Despite the considerable complexity of mobility visitations, we found that lifestyles can be automatically decomposed into only 12 latent interpretable activity behaviors on how people combine shopping, eating, working, or using their free time. Rather than describing individuals with a single lifestyle, we find that city dwellers’ behavior is a mixture of those behaviors. Those detected latent activity behaviors are equally present across cities and cannot be fully explained by main demographic features. Finally, we find those latent behaviors are associated with dynamics like experienced income segregation, transportation, or healthy behaviors in cities, even after controlling for demographic features. Our results signal the importance of complementing traditional census data with activity behaviors to understand urban dynamics.

**Supplementary Information:**

The online version contains supplementary material available at 10.1140/epjds/s13688-023-00390-w.

## Introduction

Cities are the main ground on which our society and culture develop today. Most of our current understanding of problems like transportation, mobility, inequality, gentrification, or even social participation is based on census or survey information, which is updated infrequently, contains only coarse-grain information, and is scattered across different agencies or institutions [1]. On the other hand, we now have the potential to complement official data with high-resolution updates on how people purchase, move, get a job, or interact by leveraging new sources of information from mobile data [2, 3], social media [4, 5], wifi networks [6, 7], phone apps [8, 9], and credit cards [10, 11]. Companies have been using this wealth of data in the past. They are currently able to micro-segment clients based on their demographics and their behavioral traits [12–14]. However, most cities are still using primary segments of census groups (residential areas, housing prices, gender, age, unemployment) or small behavioral surveys to map problems like inequality, gentrification, or transportation. This approach falls short of anticipating, monitoring, or forecasting the rapid and complex evolution of those problems in our cities. For example, the recent pandemic has highlighted the shortcomings of using outdated, non-integrated, and slow-processed data to manage and anticipate the spreading of COVID-19 and the special relevance of real-time, more granular, and high-frequency mobility data [15, 16].

In particular, people’s mobility data has become more available thanks to the prevalence of location acquisition techniques and mobile phones, and it enables a new way to study and understand human behavior in cities. People’s mobility behavior, e.g., the places they visit and their visiting frequency, can reflect people’s lifestyle, understood as “the way in which a person or group lives” [13, 17, 18]. Given the importance of lifestyles to predict individual and a group of individual’s behavior, they have been thoroughly explored mainly in marketing [13] but also in many fields from transportation, [19], health [20–22] to psychology and sociology [14, 23].

The study of activity patterns and detection of lifestyles of urban residents based on survey data has a long tradition, [23] but recent developments in data collection and analysis have allowed the unveiling of the high-dimensional, rapid-changing, and complex lifestyles in our cities [10, 17–19, 24–28]. Studies that try to detect those lifestyles from activity data are generally limited by the completeness of the activity/mobility space (only expenditure patterns [10], mobility patterns only when mobile phone calls and messages appear [29, 30], only transportation transit patterns [25], or a very small number of demographic variables [19]), the limited geography (only one or two cities [10, 26]), or the number of people used to detect lifestyles [17, 27]. As a result, a small number of meaningful lifestyles were detected, insufficient to accommodate the highly heterogeneous and complex variability of our cities’ behaviors.

On the other hand, in those studies, city residents’ behaviors are typically classified into a single lifestyle group [10, 19]. This forces us to divide very similar individual behaviors into different groups just based on slight differences. Consequently, a significant fraction of individuals end up with unclassified lifestyles groups [10], or across groups with minimal different characteristics [19]. These problems severely limit those lifestyle groups’ potential applicability to understanding problems like social-economic integration, mobility, or health, since slight individual behavioral differences or even different or incomplete datasets can yield a different grouping of users or lifestyles [18, 31–33].

In this work, we uncover people’s lifestyles using a dataset of mobility traces of more than 1.2 million anonymous, opted-in users in 11 cities in the United States. By formulating people’s behavior using venue and temporal activity vectors, we can extract a set of interpretable latent activity behavioral patterns [34]. Those latent behaviors are groups of visitation patterns that frequently co-occur in our sample of users. People’s lifestyle is not a label for each individual but rather a linear combination of those latent behaviors with different weights. We investigate whether those behaviors can be predicted by simple demographic traits, e.g., race, income, or transportation. Although we find a small correlation, latent behaviors seem to be primarily independent of those demographic traits. Finally, we find that each component of those latent patterns has a different relationship with social, mobility, and health problems. Our results indicate that it is possible to construct a *behavioral rich census* of lifestyles in the U.S. cities that can complement traditional census to understand the main processes and problems in our cities.

## Methods

### Mobility data

Our primary data source is from Cuebiq, a location intelligence, and measurement company that, in 2017 supplied six-month-long records of anonymized, privacy-enhanced, and high-resolution mobile location pings across 11 U.S. census core-based statistical areas (CBSAs), see Additional file 1, Supplementary Note 1. Cities are defined as the Census Core Based Statistical Areas [35] that are socioeconomically metropolitan areas related to an urban center. It consists of approximately 67 billion records from $N =1.2$ million anonymous opted-in devices, each of which has reported a total of at least 2000 locations over the six-month observation period. Our second data source is a collection of approximately 1.1 million verified venues across all CBSAs, obtained via the Foursquare API in 2017. Those venues are classified into different categories according to the Foursquare Category Hierarchy [36]. Only users with more than 50 visits during the period and with at least 5 categories visited were considered. We only considered the top most visited 248 categories to prevent over-fitting to small, infrequent categories. We infer the *home area* of each individual at the Census Block Group level using their most common location between 10 pm and 6 am. We further extract any individual visits to a given place that lasts for more than 5 minutes (see Additional file 1, Supplementary Note 1) and are less than 4 hours long. We have also tested that our results do not depend on this definition of visits to POI, see Additional file 1, Supplementary Note 1. It is important to note that our visitation patterns include not only consumption patterns (restaurants, shops, sports events, etc.) but also other non-commercial activities (transportation, education, health, outdoor activities, etc.), which are important to explain urban lifestyles.

### Demographic data

Due to the anonymous nature of our location data, we obtained the demographic characteristics of each user at the area level. Demographic data like median household income, the fraction of Black population, the fraction of people that use public transportation, or urban characteristics like population density were obtained from the Census 2013-2017 ACS 5-year Estimates [37]. The fraction of people with more than 45 minutes of commuting was also obtained from the ACS Data.

### Social, transportation and health data

To assess the importance of the latent behaviors, we validate them using different social, transportation and health data. The source of transportation data is the 2017 Local Area Transportation Characteristics for Households Data done by the Bureau of Transportation Statistics (BTS) [38], while obesity prevalence and physical activity are given by the 500 Cities Project Data from the Center for Disease Control (CDC) [39]. Obesity prevalence is measured as the percentage of adults, aged 18 or older, who report a body mass index (BMI) of 30 or higher. Physical activity is measured as the fraction of adults who report getting leisure-time physical activity in the past month. Cities are defined as the Census Core Based Statistical Areas [35] that are socioeconomically metropolitan areas related to an urban center. Note that although we could have constructed the mobility variables using our data, we used the BTS and Census data because the latter is based on more reliable estimation statistics. But also because our data do not have complete daily individual trajectories of people, preventing us from having a precise estimation of the distance traveled and the commuting time.

To measure experienced social-economic integration, we use the inequality metric introduced in [8] to estimate how unequal is the exposure of an individual to the different income groups in the city. To this end, we divide the sample of users in each city into four quartiles according to the median household income of their home Census Block group [37]. Social-economic integration was measured as $I_{i} = 1- \frac{2}{3}\sum_{q}|\tau _{iq}-1/4|$, where $\tau _{iq}$ is the proportion of time user *i* is exposed to group *q* of income. That proportion is calculated by looking at the weighted distribution of income of the people that *i* encounters in the places she visits. Specifically $\tau _{iq} = \sum_{\alpha }\tau _{i\alpha}\tau _{q\alpha}$, where $\tau _{i\alpha}$ is the fraction of time that *i* spends at place *α* and $\tau _{q\alpha}$ represents the proportion of time at place *α* spent by income group *q*. Our metric for individual economic integration can be thought of as an extension of the traditional metric of isolation or interaction for groups to the level of individuals based on daily encounters among them. Finally, social exploration is measured as $E_{i} = S_{i} / N_{i}$, where $S_{i}$ is the total number of different places visited by *i* and $N_{i}$ is the total number of visits to places by *i*. See [8] for more details on these metrics and their distribution.

### Non-negative matrix factorization

**Figure 1:**
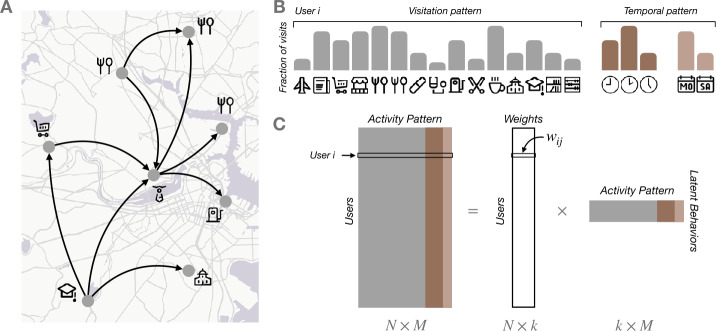
*Detecting latent behaviors* (**A**) Using individuals’ trajectories, we identify the visits to the different places and the categories of those places. (**B**) Each individual is described by a *M*-dimensional (normalized) vector that contains the fraction of visits to each of the 286 categories (visitation pattern) plus the fraction of visits at different times during the day and the week (temporal pattern). (**C**) Non-negative matrix factorization is used to decompose the matrix of the *M*-dimensional vectors for each of our *N* users into a matrix of *k* different latent behaviors and the corresponding behavior’s weights for each user. Icons designed by bqlqn/flaticon.com and Boston maps produced using Open Street Map data

Rather than describing a person by a unique pattern, we will assume that there are some latent behavioral patterns that, when combined, define a person’s lifestyle. The weight of the different latent behavior patterns could reflect their dominance over the person’s lifestyle. To detect those latent behavioral patterns, we describe the activity of each user *i* by a *M* dimensional vector, which includes the (normalized) number of visits to the different types of places (248 venue categories, see Fig. 1 and Additional file 1, Supplementary Figure S2). We also include five temporal features about the fraction of those visits that happen during the morning (5 am to noon), afternoon (noon to 6 pm), evening/nighttime (6 pm to midnight), and also during the weekend and weekdays. Thus user *i* activity is described by a vector $\mathbf{x}_{i}$ of $M = 248+5$ components. As we will see in the results, adding the temporal pattern allows to recover different latent behaviors by day of the week.

When put together for all *N* users, they form a matrix *X* of $N \times M$ dimensions. Different methods exist to learn the latent patterns for the vectors of those *N* users, like spectral methods [3], Latent Dirichlet Allocation [25, 27, 29], neural networks, [17] or complex networks [10]. In general, these methods detect latent patterns, i.e. co-occurrence of variables (visitations in our case) that frequently appear in the dataset. Given that our vectors are non-negative, we apply non-negative matrix factorization (NMF) to *X*. NMF is a powerful technique for finding parts-based, linear representations of non-negative data and has been applied successfully in several applications like genomics, image recognition, or text mining [40, 41]. In the context of human behavior, it has also been used to identify the activity patterns of users or urban areas [26, 30, 42–44].

The key idea is that the activity matrix can be decomposed into two matrices $X = W \cdot B$, where *B* is a matrix of dimension $k \times M$ that contains each of the *k* latent behaviors and *W* is a $N \times k$ matrix that contains the weights of those latent behaviors for each user (see Fig. 1(C)). Thus each $\mathbf{x}_{i}$ can be decomposed as linear combination of *k* latent behaviors $\mathbf{x}_{i} = \sum_{j=1}^{k} w_{ij} \mathbf{b}_{j}$. In each latent behavior pattern $\mathbf{b}_{j}$, the higher value the type of venue or temporal feature holds, the more dominant that category of place or time of the week acts in the latent behavior pattern. For each user *i*, the higher the $w_{ij}$, the more important is latent behavior $\mathbf{b}_{j}$ to explain her activity.

The activity matrix *X* is factorized using different ranks *k*. To do that, we used non-negative matrix factorization (NMF) using fast sequential coordinate-wise descent and Kullback–Leibler (KL) divergence for the loss function. We run the NMF two hundred times for each value of *k*. Different methods [30, 44] were used to assess the value of *k* (see Additional file 1, Supplementary Note 4), including bi-cross validation [45]. We found that $k = 12$ was the one that optimizes the error, and the stability of the weights *W* and the bi-cross validation error, while making the latent behaviors *B* more interpretable across realizations. For that $k=12$, we chose *B* and *W* from the realization with smaller KL loss (see Additional file 1, Supplementary Note 4). Our NMF factorization also produces very close representations of the whole mobility data. As we can see in Additional file 1, Supplementary Note 4, the KL average distance $d_{KL}(X,W\cdot B) = 0.0195 \pm 0.0001$ or the Frobenius distance $d_{F}(X,W\cdot B) = 0.0033 \pm 0.00004$ between the original data and the reconstructed one are very small. This means that on average, the error of our approximation is below 15% relative error.

To prevent an over-representation of the larger cities in the factorization, we did not use our 1.2 million users in the NMF. Instead, we randomly selected 10k users in each city and constructed the matrix $X_{0}$. We factorize it into $W_{0} \cdot B_{0}$ and use $B_{0}$ to solve the non-negative linear regression problem $X \sim W \cdot B_{0}$ to get *W* for the rest of the users. This way, we get a fair representation of all the latent behaviors commonly present across cities. In any case, we have also checked that our results are robust against different definitions of the sample of users, see Additional file 1, Supplementary Note 7. To compare with other methods to detect latent patterns, we have also used LDA to detect latent behavior patterns (see Additional file 1, Supplementary Note 5). Although the results are somehow similar, we find that the latent behavior patterns detected by NMF have better interpretations than those from LDA.

## Results

### Latent behaviors

**Figure 2:**
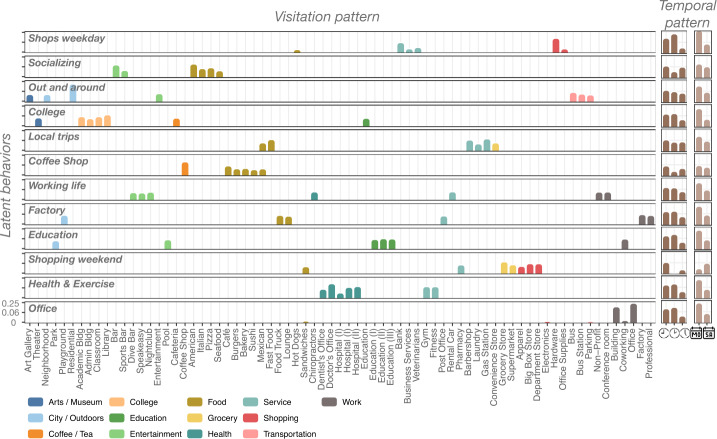
*Latent behaviors:* Visitation and temporal components for each of the $k=12$ latent behaviors detected. For simplicity, only the top 7 venue category components by behavior are shown. Colors correspond to the different classifications of the venues. Temporal patterns correspond to the fraction of morning, afternoon, and night visits together with the fraction of weekday and weekend visits. The “Out and around” main components are residential and bus transportation during weekdays, while “Socializing” is composed of visitations to bars, sports bars, and food places like Italian, American, pizza, or seafood, especially during the evenings/nights. Finally “Local Trips” refers to a latent behavior mainly composed of running errands (laundry, gas station, convenience store) and visitation to fast food or food truck venues

The moderately large number of latent behaviors shows the richness and heterogeneity of our dataset. To interpret the latent behavior patterns, we first look into the different categories’ dominance values and time slots (see Fig. 2). As we can see, most of the latent behaviors are easily recognizable and, as expected [26], their most relevant components belong, generally speaking, to combinations of working, food, entertainment, or shopping activities. Note that they are not strict projections only on one of those dimensions. For example, we find a latent behavior (“Working life”) of working-related activities (Conference Room, Non-Profit) and nightlife venues, or a latent behavior “Out and around” that combines public transportation (bus) with neighborhood visits. Our choice of including the daily and weekly temporal pattern allows us to detect even different shopping behaviors between weekends (“Shopping weekends” that also includes grocery shopping) and weekdays (“Shops weekdays”). Other distinct latent behaviors correspond to “College” students, “Coffee shop” frequenters, or “Health & Exercise” visitors. Note that our denomination of the latent behaviors is based on the most dominant categories, which are also the most visited categories in cities. This does not mean that other less-visited categories are not part of those behaviors. For example, most latent behaviors have some components in the Food category (see Additional file 1, Supplementary Figure S2). However, their relative importance is smaller than in the “Local trips”, “Coffee Shop” or “Bar + Food” latent behaviors. Nevertheless, even for the places that are visited less frequently in our cities, the NMF can detect distinct patterns there. For example, the “Coffee Shop” latent behavior has large components in airport transportation venues than the rest of the behaviors (see Additional file 1, Supplementary Figure S2). Finally, it is worth noticing that our detected latent behaviors are not only related to expenditure: an analysis based only on expenditure patterns would have probably missed important latent behaviors like “Out and around”, “Office”, “College” or “Education”.

By definition, our latent behaviors encode those visitation patterns which are more frequently happening together. For example, we find in the “Local trips” latent behavior that fast food consumption is related to local errands, while nightlife is associated with work-related places like Conference Rooms or Rental Cars. “Shopping weekend” tells us that people tend to bundle visits to pharmacies, retail, groceries or departmental stores together. At the same time, in “Coffee Shop,” we see that visits to coffee shops are associated with the consumption of some types of food like Bakery, Sushi, and Burgers. Thus, our latent behaviors also tell us how people organize their mobility visitation patterns in their daily lives.

**Figure 3:**
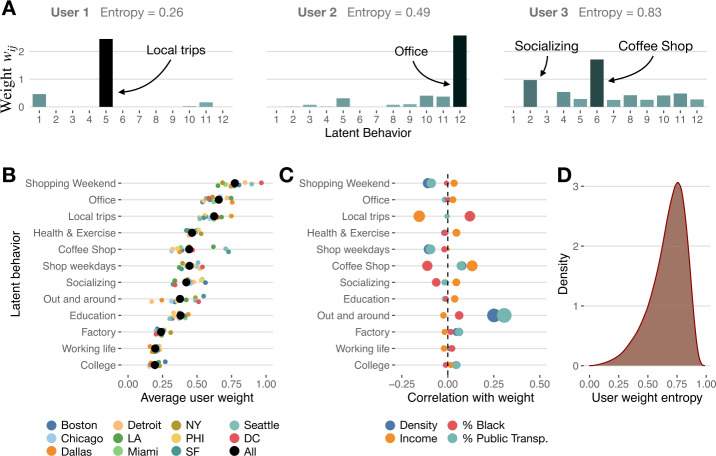
*Users’ latent behaviors and lifestyles* (**A**) Distribution of weights by latent behavior for three different users in our database. User 1 behavior is dominated by the “Local trips” behavior and has low entropy. However, user 3 has a larger entropy, and her behavior is a very diverse mixture of latent behaviors, including “Bar + Food” and “Coffee Shop”. (**B**) Average weight for the different latent behaviors in all areas (black) and different cities. (**C**) Correlation between the weight of latent behaviors and different demographic and urban characteristic features like population density, median income, percentage of Black population, and percentage of people using public transportation. (**D**) Distribution of user’s weight entropy

As we said, each person’s activity lifestyle can be described as a linear combination of the latent behavior patterns according to their weights obtained by NMF (see Fig. 3). Instead of being described as a simple latent behavior, we find that a user’s lifestyle generally depends on many behavior patterns. This is shown by the large entropy of weights by user $S_{i} = -\sum_{j=1}^{k} \hat{w}_{ij} \log \hat{w}_{ij} / \log k$ (where $\hat{w}_{ij}$ is the normalized weight by user), see Fig. 3(c). Strict dominance of single latent behavior would make $S_{i}=0$, while we get $S_{i}=1$ if all latent behaviors are present and equally important. In our data, we obtained that the average entropy is $\overline{S}_{i} = 0.67 \pm 0.15$, and thus many latent behaviors configure each user’s activity pattern.

However, some latent behaviors are, in general, more frequent than others. For example we find that the average weight of “Shopping Weekend” ($\overline{w}_{ij} = 0.774 \pm 0.001$) or “Office” ($\overline{w}_{ij} = 0.657\pm 0.001$) is larger than others like “Working life” ($\overline{w}_{ij} = 0.197 \pm 0.001$) or “College” ($\overline{w}_{ij} = 0.195 \pm 0.001$), which signals that, as expected, the former latent behaviors are more common in urban areas than the latter ones. That is, most individuals have a very small (or even zero) “College” latent behavior, while most people have some weight on “Shopping Weekend” behavior.

Interestingly, we find that these results are pretty robust across all the cities studied (see Fig. 3(B)), which means that each latent behavior’s relative weights are very similar despite the different geography, density, or even cultural nature of the cities. This is an important result that demonstrates our method’s robustness across different cities and the homogeneity of activity patterns across the U.S. However, there are small but important variations. For example, the relative weight of the “College” behavior is larger in Boston $\overline{w}_{ij}^{\mathrm{Boston}}= 0.270\pm 0.001$ because of the large population of university students in the area. Also, cities with better public transportation systems like Boston, Washington DC, or N.Y., have larger weights in the “Out and around” latent behavior than cities like Dallas or Detroit, where public transportation is scarce. Other significant variations happen in the “Coffee Shop” latent behavior, more present in cities like San Francisco or Seattle than in the rest. This is expected given that those cities are the ones with the most coffee shops per capita [46]. Taking together these results shows the adaptability and robustness of our latent behaviors to describe the similarities and peculiarities of activity patterns in the different cities in the U.S.

### Latent behaviors are not fully described by demographics or urban characteristics

One important question is whether the detected latent behaviors can be explained by individuals’ simple demographic or urban characteristics. Group segmentation using census-type only data is traditional in marketing, [12, 47] and even the U.S. Census Bureau used it to design its 2020 campaign [48]. Since mobility is highly influenced by socioeconomic status, access to public transportation, or the density of the urban area, we could expect that the detected latent behaviors might depend strongly on those features. However, we find that users’ weights $w_{ij}$ are largely independent of the median income, population density, the fraction of Black population, or even the fraction of people using public transportation (see Fig. 3(C) and Additional file 1, Supplementary Table S1). We only find moderate correlations with some latent behaviors. For example, low-income people have more “Local trips” latent behavior, while “Coffee Shop” is a behavior more likely to be found in high-income areas. Of course, “Out and around” latent behavior is more prevalent in areas with higher public transportation use. Apart from those cases, the correlation between our latent behaviors’ weights and demographic and urban features is very small $R^{2} \leq 0.1$ (see Additional file 1, Supplementary Note 6 and Additional file 1, Supplementary Table S1). Thus, latent behaviors detected using mobility data are different from the traditional census demographic traits. The detected activity patterns give a different and complementary perspective of our cities than traditional census analysis, allowing us to construct a richer *behavioral census* that includes those behaviors.

### Association of latent behaviors with social, mobility, and health problems

To demonstrate the complementary power of the latent behaviors to traditional census approaches, we have analyzed their association with different social, mobility, and health outcomes in the 11 cities. In the social dimension, we have considered $I_{i}$, the income integration (or diversity) experienced by each individual, introduced in [8]. This quantity reflects how homogeneous is the exposure of each individual to the different income groups in the city: by using the household median income of the Census Block group where user *i* lives, we can quantify the income group (income quartile within each city) she belongs to. Using that information for each user, we can estimate the amount of time a user *i* is exposed to the different income groups in the city while visiting different venues: if $I_{i} = 0$, individual *i* only goes to places where her particular income group is the majority. If $I_{i} = 1$, the individual is exposed equally to people from all the city’s income groups (Methods Sect. 2.3). Other versions of diversity exposure have been analyzed recently [9] and, in particular, income exposure diversity is related to social capital and impacts economic opportunities, and social income mobility of individuals [49]. Also, along the social dimension, we have studied the individual’s place exploration $E_{i}$, which measures the rate of visitation to different places by *i* in our time period [50]. That is, if $N_{i}$ is the number of visits made by user *i* and $S_{i}$ is the number of unique places visited, then $E_{i} = S_{i} / N_{i}$. Although people spend most of their time in a very small number of places [2, 24], it is well known that some tend to visit more places (*explorers*, $E_{i} \simeq 1$), while some others spend most of their time in a small set of places (*returners*, $E_{i} \simeq 0$). Explorers are people that go very often to never visited before places, while returners are constantly coming back to places that they already visited. Those two distinct classes of individuals have been found in many different mobility studies [2, 8, 51], but also in other people’s activities like social network connections [52], web browsing [53], or knowledge discovery [54].

Both income exposure diversity and place exploration are crucial to understanding the social component of mobility in our cities and, specifically, how segregated (not integrated) people are. As was found in [8] experienced income integration is moderately and positively related to place exploration ($\rho = 0.456 \pm 0.001$). To test the association of the latent behaviors in these problems, we have used a regression model:

1 $$\begin{aligned} I_{i}, E_{i} \sim \sum _{j=1}^{k} \beta _{j} w_{ij} + \sum_{l=1}^{m} \gamma _{l} d_{l} + \mathrm{MSA}_{i} + \varepsilon _{i}, \end{aligned}$$

where $d_{l}$ refers to the four demographic and urban features mentioned before (median household income, the density of the area, the fraction of Black people, and the fraction of use of public transportation), and $\mathrm{MSA}_{i}$ is a fixed factor by city (Metropolitan Statistical Area). Including the census variables and city-fixed effects allows us to investigate the fundamental role of latent behaviors once we control for potential effects by demographic and urban characteristics and the city where users live. In our models, census features are always less important to explain that variability ($R^{2} = 0.059$ for $I_{i}$ and $R^{2} = 0.025$ for $E_{i}$ only using census variables) than our latent behaviors ($R^{2} = 0.164$ and $R^{2} = 0.26$ respectively using also latent behaviors, see Additional file 1, Supplementary Tables S2 and S3. This result shows that our latent behaviors encode most of the social-economic integration and exploration variability across users, which are largely independent of census variables.

**Figure 4:**
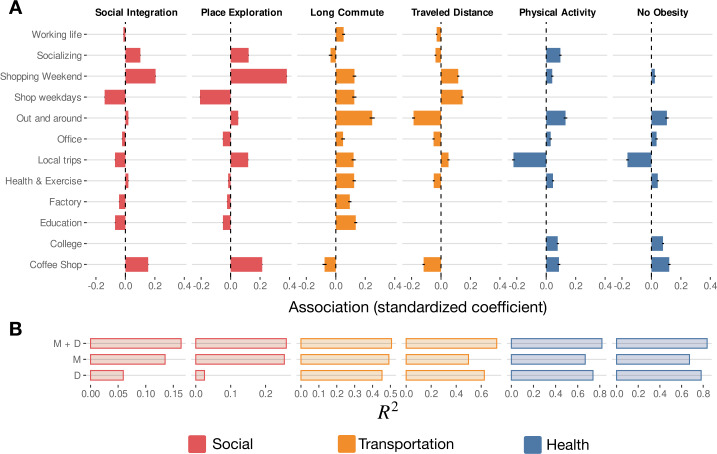
*Relationship of behavioral patterns with social, mobility, and health problems*. (**A**) Bars show the coefficient of each behavior weight (columns) on the different regression models for each social-economic integration, place exploration, the fraction of people with longer than 45 min commute, amount of distance traveled by day, the fraction of people doing daily physical activity, and the fraction of people not having obesity (rows). (**B**) Bar shows the $R^{2}$ for each model, including only the demographic variables *D*, only the mobility behavioral weights *M*, and both together $M+D$. Panel (**A**) correspond to the coefficients for the $M+D$ case. See Additional file 1, Supplementary Note 6 for the complete regression tables

Nevertheless, not all latent behaviors have the same effect on experienced income segregation and exploration. Figure 4 shows the relationship [measured as the standardized coefficient $\beta _{j}$ in the model (1)] of the different latent behaviors with both social problems. We find that the latent behaviors that impact economic integration are related to shopping or food/coffee. In contrast, others like college, office, or health are not heavily associated with economic integration. Interestingly, behaviors like “Shopping Weekend” or “Coffee Shop” are positively associated with economic integration, while behaviors “Education”, “Factory” or “Shopping weekdays” are more present in users with more considerable experienced income segregation. This might be explained by the fact that shops, coffee shops, or some restaurants are more economically diverse in the city than factories, education, or local shops [8]. In general social exploration follows the same pattern, although people with more “Local trips” behavior tend to be more explorers without being more integrated. These results show that the latent behaviors carry significant explanatory power of the income diversity and exploration experienced by people in the urban areas analyzed.

We have also studied other problems related to transportation and health. Lifestyles are crucial to understanding our mobility choices or opportunities and transportation routines [19], but also physical activity and the prevalence of some health conditions [21]. Since we do not have individual health conditions, we have used data from the Census, the Bureau of Transportation Statistics (BTS), and the Center for Disease Control to take variables by census tract *α* describing the average distance traveled by residents $D_{\alpha}$, the fraction of people that have more than 45 minutes of commuting $C_{\alpha}$, the fraction of people with leisure-time physical activity in the past month $P_{\alpha}$ and the fraction of people with no obesity $O_{\alpha}$ (see Methods Sect. 2.3). To study the relationship of our latent behaviors with those problems, we construct the average weight by census tract $\hat{w}_{\alpha,j}$ for all the users living in that tract *α* and fit them using similar models as Eq. (1), see Additional file 1, Supplementary Note 6.

As we can see in Fig. 4, local behaviors like “Out and around” have a strong negative association with the distance traveled, although the association with commuting duration is positive. In general, tracts with more shopping behavior travel more, while those with larger “Coffee Shop” or “Bar + Food” latent behavior have smaller commutes and distance traveled. Since our data is projected at the level of the census tract, individual variability is averaged out, and thus, demographic and urban variables are more important to explain the variability in transportation variables. However, our latent behaviors still explain part of how people commute or move around the city, even if we condition on income, race composition, density, or use of public transportation (see Additional file 1, Supplementary Note 6).

Finally, we see in Fig. 4 that, although not all of them are relevant, there are a fraction of latent behaviors that have a significant association with health outcomes. As expected, more presence of latent behaviors like “Local trips” (which include visits to Fast Food venues) is associated with less Physical Activity and more Obesity, while behaviors like “Out and around” or “Coffee Shop” are positively associated with the amount of Physical Activity and the absence of Obesity. However, social and mobility important behaviors like shopping are not significantly related to health outcomes. This is even after controlling for demographic variables like income or race, which are the most critical determinants of those health outcomes [55].

In summary, our results show that our latent behaviors that constitute individual lifestyles are significantly correlated with social, transportation, and health outcomes. Different behaviors are related to different dimensions, and in some cases, the importance of latent behaviors is similar to or even surpasses that of demographic variables. For example, knowing that a particular user has extensive shopping or food/coffee behaviors can better explain her economic integration and exploration than knowing her income (see Additional file 1, Supplementary Tables S2 and S3). Or conditioning on income or population density, we see that some behaviors like “Out and around” or “Coffee Shop” are associated with transportation and health outcomes.

## Discussion

Understanding urban problems require a good description of human behavior in cities [1, 56]. Our research shows that the high-dimensional nature of mobility of millions of people visiting millions of places in the U.S. can be projected onto a small set of latent behaviors that capture their routines and habits that result from their choices or opportunities accessible to them. Demographics or urban characteristics cannot fully explain those latent behaviors, and people living in the same neighborhood with the same income, race, or educational levels might have different shopping, working, or leisure latent behaviors, resulting in entirely different lifestyles. Since the composition of the lifestyles is robust across different geographical areas and cities, our results could be used to build characterizations and compare individuals and groups at different geographical and demographic levels. This could enrich the current Census by including the composition of the different latent behaviors to study urban areas. It could also help in methods of exploiting mobility data by preserving the privacy of individuals. This can be done by computing projected aggregated variables along those latent behaviors rather than detailed and more invasive individual visitation patterns.

Our latent behaviors describe how people organize their visitation patterns and mobility around the city. For example, we find that people that make trips to errands also visit fast food outlets frequently (“Local trips” latent behavior), while heavy users of public transportation (bus) also spend much time in the neighborhood and entertainment (“Out and around”). Working life is also related to nightlife. These dependencies show that those latent behaviors represent combined aspects of our life that occur concurrently and which could be used to devise successful holistic interventions to change people’s lifestyles. For example, people that run many errands might choose fast food because they are time-poor or because errands take place around specific food environments (food swamps). Our results can help design public health interventions that incorporate those distinct lifestyles to identify those routines and habits that are most risky for health [57].

We note that latent behaviors have a different relationship with social, transportation, and health outcomes. For example, while weekend shopping behaviors are associated with more exposure to economic diversity of urban dwellers, they carry more commuting time and travel distance and, thus, more pollution. Similarly, the “Out and around” latent behavior is associated with longer commutes and more physical activity or the absence of obesity. Since most urban interventions are likely to change the relative weight of those latent behaviors or ultimately change them completely, it is essential to balance the trade-off among social, transportation, and health outcomes encoded in those behaviors. Also, not all behaviors have the same weight in describing users’ lifestyles. Shopping, food, or working latent behaviors are the most important, suggesting that they are the ones where interventions to change experienced income segregation, transportation, or health outcomes could be more considerable [25].

Our results show that activity lifestyles are not monolithic groups of homogeneous behavior among people. Our framework of describing lifestyles as a combination of latent behaviors reflects that lifestyles are instead a continuum spectrum of the relative balance between work, shopping, transportation, or leisure time. Given the ubiquitous nature of mobility and activity data from mobile phones, we hope this framework could be used in the future to understand better the rapid and extensive scale changes in other urban areas and cities worldwide.

## Supplementary Information

Below is the link to the electronic supplementary material.

Additional file contains Supplementary Note 1—Data, Supplementary Note 2—Representativity, Supplementary Note 3—Non-negative matrix factorization, Supplementary Note 4—Rank Selection, Supplementary Note 5—Comparison with LDA, Supplementary Note 6—Models, and Supplementary Note 7—Robustness checks. It also contains Supplementary Figures S1 to S5 and Supplementary Tables S1 to S4. (PDF 838 kB)

## Data Availability

The data that support the findings of this study are available from Cuebiq through their Data for Good program, but restrictions apply to the availability of these data, which were used under license for the current study, and so are not publicly available. Aggregated data used in the models are however available from the authors upon reasonable request and with permission of Cuebiq. Custom code that supports the findings of this study is available from the corresponding author upon request.
