# Supplementary material for: Identifying latent activity behaviors and lifestyles using mobility data to describe urban dynamics
Source: EPJ Data Sci. 2023 May 18;12(1):15. doi: 10.1140/epjds/s13688-023-00390-w (PMC10193357; doi:10.1140/epjds/s13688-023-00390-w)
Supplement: Supplementary file 1 — Additional file contains Supplementary Note 1—Data, Supplementary Note 2—Representativity, Supplementary Note 3—Non-negative matrix factorization, Supplementary Note 4—Rank Selection, Supplementary Note 5—Comparison with LDA, Supplementary Note 6—Models, and Supplementary Note 7—Robustness checks. It also contains Supplementary Figures S1 to S5 and Supplementary Tables S1 to S4. (PDF 838 kB) [file 13688_2023_390_MOESM1_ESM.pdf]

Supplementary Material for
Identifying latent activity behaviors and lifestyles using mobility data to describe urban dynamics

Yanni Yang, Alex Pentland, Esteban Moro*

*Corresponding author: esteban.moroegido@gmail.com

Supplementary Notes

1 Data **2**

2 Representativity **2**

3 Non-negative matrix factorization **2**

4 Rank selection **3**

5 Comparison with LDA **4**

6 Models **4**

7 Robustness tests **5**

7.1 Duration of visits 5

7.2 Sample of users 5

1 Data

The mobility data were obtained from Cuebiq, a location intelligence and measurement company. The dataset consists of anonymized records of GPS locations from users that opted-in to share the data anonymously in the Boston metropolitan area over a period of 6 months, from October 2016 to March 2017. Data was shared in 2017 under a strict contract with Cuebiq through their Data for Good program, where they provide access to de-identified and privacy-enhanced mobility data for academic research and humanitarian initiatives only. All researchers were contractually obligated not to share data further or to attempt to de-identify data. Mobility data is derived from users who opted in to share their data anonymously through a General Data Protection Regulation (GDPR) and California Consumer Privacy Act (CCPA) compliant framework.

From the data, we extracted the "stays" as the places where anonymous users stayed (stopped) for at least 5 minutes using the algorithm proposed by Hariharan and Toyama [1]. Some of the stays happen within places (Points of Interest). We use a dataset of 1.2 Million Points of Interest in US metropolitan areas collected using the Foursquare API. We use the Foursquare venue categorization of the places to detect the type of place visited [2]. Finally, we estimate the home Census Block Group of the anonymous users as that in which they are more likely located during nighttime. This results in a dataset of the places people stayed, including the points of interest that anonymous users visited and the most likely census block group of where the device owner lives.

We only consider stays that last more than 5 minutes for several reasons: first and most importantly, shorter stays cannot be detected with enough precision by our algorithm [1]. That threshold is similar to other implementations of that algorithm [3]. Furthermore, we want to discard people just passing by or stopping for a short time close to the food place. On the other hand, we consider only visits to a given place (not home) shorter than 4 hours to discard stays related to working places. That is because some of our users are not visiting a venue but rather working there. Considering working places will produce an over-representation of some categories (their working place) for each user. Thus rather than lifestyles, our method would detect just jobs. Finally, with this upper threshold, we only discarded 5.49% of the visits to different places that lasted for more than 4 hours. However, our main results are not affected by this particular threshold (see 7). Finally, to perform the matrix factorization, we have only considered users with more than 50 visits and visited more than five places categories.

We only considered mobility data that happen within 11 metropolitan areas defined as the Core-based Statistical Areas (CBSA) [4]. We considered CBSAs instead of other geographical units, since they are areas that are socioeconomically related to an urban center. This provides a self-contained metropolitan area in which people move for work, leisure or other activities. Note that most of the CBSAs we consider span several states.

2 Representativity

Our location data comes from smartphones in large urban areas. Although a large proportion of the U.S. population owns a smartphone in urban areas, we might question whether our sample of users is representative of the population and different socio-demographic groups in that area. The correlation between the population of our users and the one from the census is high ($\rho = 0.65 \pm 0.01$), showing that we get a good representation of the population. A more extensive comparison of the demographic representativity of our sample of users was made in reference [5], where we saw that our sample was slightly biased towards high-income people. Note, however, that some of our results are largely independent of this bias, since our models are trained at individual level, considering income as part of the regression model (Figure 4 on the main paper).

3 Non-negative matrix factorization

Non-negative matrix factorization is a well-known technique to approximate a non-negative matrix $X \in [0, \infty]^{m \times n}$ using non-negative low-rank matrices $W \in [0, \infty]^{m \times k}$ and $H \in [0, \infty]^{k \times n}$ such that $X \simeq WH$. This is typically achieved by looking for the W and H that minimize a loss function. Typical choices are the quadratic loss

$$d_F(X, WH) = ||X - WH||_F^2, \tag{1}$$

where $||\cdots||_F$ is the Frobenius norm, or the Kullback-Leibler divergence distance $d_{KL}(X, WH) = \mathrm{KL}(X, WH)$, where

$$\mathrm{KL}(A, \hat{A}) = \sum_{ij} a_{ij} \log \frac{a_{ij}}{\hat{a}_{ij}} - a_{ij} + \hat{a}_{ij}. \tag{2}$$

Different algorithms exist to approximate the minimum of the loss function to get W and H. In our case, we have used the fast sequential coordinate-wise descent introduced by Lin, and Boutros [6] and implemented in the `NNLM` package in `R` [7]. For the loss function, we used the Kullback-Leibler divergence metric.

Note that since the non-negative matrix factorization is not guaranteed to be unique, each factorization slightly depends on the different initial conditions. Thus, for each rank k we have run 200 realizations. For presentation purposes, the realization with the smallest error by k was used in the definitions of the latent behaviors weights w_{ij} used here and in the main paper.

4 Rank selection

There are several strategies to choose the rank k in the non-negative matrix factorization (NMF). As in other latent detection methods, typically, a combination of statistical and interpretability criteria is used [8, 9, 10]. Popular statistical methods include observing the variation of the residual sum of squares (RSS) between the original matrix and its factorization [11] or measuring the stability of the weights in the NMF [12] across different realizations. Other state-of-the-art methods include bi-cross validation methods [13], where a set of rows and columns are left out to evaluate the goodness of the factorization to reconstruct them. But we also would like to get latent behaviors that are interpretable, that is, that can be described by a number of significant components (not too sparse) and have some meaning according to human interpretation.

In our case, we have chosen a combination of the two approaches. To measure the sparseness of each latent behavior $\mathbf{b}$, we have used the entropy

$$S(\mathbf{b_j}) = -\frac{1}{M} \sum_{l=1}^{M} \frac{b_{jl}}{||\mathbf{b}_j||_1} \log \frac{b_{jl}}{||\mathbf{b}_j||_1}. \tag{3}$$

Entropy is zero if $\mathbf{b}_j$ only contains a non-zero component and one if all components are equal. For each k we have computed the average of all behaviors and all realizations. Although sparsity of W and B can be fixed by imposing some additional constrains in the factorization process [14], we have preferred not to used them to simplify the process of rank selection by minimizing the number of choices made and parameters used in the factorization.

For the bi-cross validation, we have used the method by Owen *et al.* [13]. In that method, a set of randomly selected r rows and s columns are removed from the original matrix X. Rearranging the matrix in the following form

$$X = \begin{pmatrix} A & B \\ C & D \end{pmatrix} \tag{4}$$

where A is the $r \times s$ submatrix. Assuming that $D = W_D H_D$ is the rank k non-negative factorization of D, we can use approximated the substracted matrix A by

$$\hat{A} = U_B V_C \tag{5}$$

where U_B is the solution of the non-negative least squares problem $U_B = \arg\min_W \mathrm{KL}(B|WH_D)^2$ and $V_C = \arg\min_H \mathrm{KL}(C|W_D H)^2$. Then the held-out error estimate for the cross-validation is $\mathrm{KL}(A|\hat{A})$. We have repeated this bi-cross validation process for 300 random selections of r rows and s columns. Specifically, we have left 10% of the rows and columns in each random selection.

Finally, to measure the stability of the components, we have used a variation of the cophenetic correlation coefficient proposed by Brunet et al. [12]. For each realization, we assign each user i to the cluster given by her largest component w_{ij} and compare those assignments across different realizations using the normalized mutual information (NMI) measure proposed by Danon et al. [15]. Finally, we average that NMI over all possible comparisons

between realizations. If all realizations give, the same clusters, then our metric will be one. However, if clusters are totally different across realizations, the NMI will be zero.

Figure S1 shows the variation of the (average) loss (KL divergence) and entropy of the latent behaviors as a function of NMF factorization ranks k. Although the KL loss curve does show only a small inflection point around $k = 12$, we can clearly see that the sparseness of the latent behaviors has a local minimum of around $k = 12$. We also find that the consensus NMI metric has a maximum at $k = 10$ and local one at $k = 12$. Finally, the bi-cross validation seems to have a local minimum around $k = 11, 12$. Given these results and the good interpretability of the latent behaviors, we chose $k = 12$ as the factorization rank in our NMF. Similar protocols to choose the rank have been used in similar datasets recently [9, 10].

5 Comparison with LDA

We have studied also the detection of latent behaviors using other methods like Latent Dirichlet Allocation (LDA). As we can see in Figure S3, the topics detected using LDA on the matrix X_0 are similar to the ones obtained using NMF. However, some of the behaviors in the NMF get mixed in the LDA, specially the ones around shopping, school or health, which make them less interpretable. Apart from these differences, this result shows that our detected latent behaviors are genuinely present in the data and that different methods yield more or less the same set of them.

6 Models

To detect the dependence between our latent behaviors, demographic variables, and social, transportation, and health outcomes, we have used Ordinary Least Squares (OLS) regressions between the variables at the individual or census tract level. In particular:

- To test the independence between the latent behaviors and the demographic variables, we have model w_{ij}, each individual i weight on latent behavior j as

$$w_{ij} \sim \text{density}_i + \text{income}_i + \text{public\_transportation}_i + \text{black\_population}_i + \varepsilon_i \tag{6}$$

  where each demographic variable is the estimation from the 2013-2017 American Community Survey 5-year Estimates [16] for the Census Block Group, where we estimate where the user lives. Results for this OLS regression are presented in Table S1, where we can see that the R^2 is clearly very small.

- To test the impact of latent behaviors on social outcomes, we have used the OLS regression at the individual level:

$$X_i \sim \sum_{j=1}^{k} \beta_j w_{ij} + \text{density}_i + \text{income}_i + \text{public\_transportation}_i + \text{black\_population}_i + \varepsilon_i \tag{7}$$

  where X_i is each individual's social integration or exploration. Results are presented in Table S2.

- Finally, since we can only access transportation and health outcomes at the Census Tract level, we test the impact of latent behaviors on those outcomes using OLS at census tract α.

$$X_\alpha \sim \sum_{j=1}^{k} \beta_j \hat{w}_{\alpha j} + \text{density}_\alpha + \text{income}_\alpha + \text{public\_transportation}_\alpha + \text{black\_population}_\alpha + \varepsilon_\alpha \tag{8}$$

  where X_α is each of the variables studied (no obesity, physical activity, long commutes or distance traveled) by census tract α and $\hat{w}_{\alpha j}$ is the average of w_{ij} for all the users living in census tract α. Results are presented in Table S2

Finally, we have built also models for X_i and X_α using only demographic variables and mobility behaviors to test the relative importance of the latent behavior weights. Results are presented in Table S3 and Table S4.

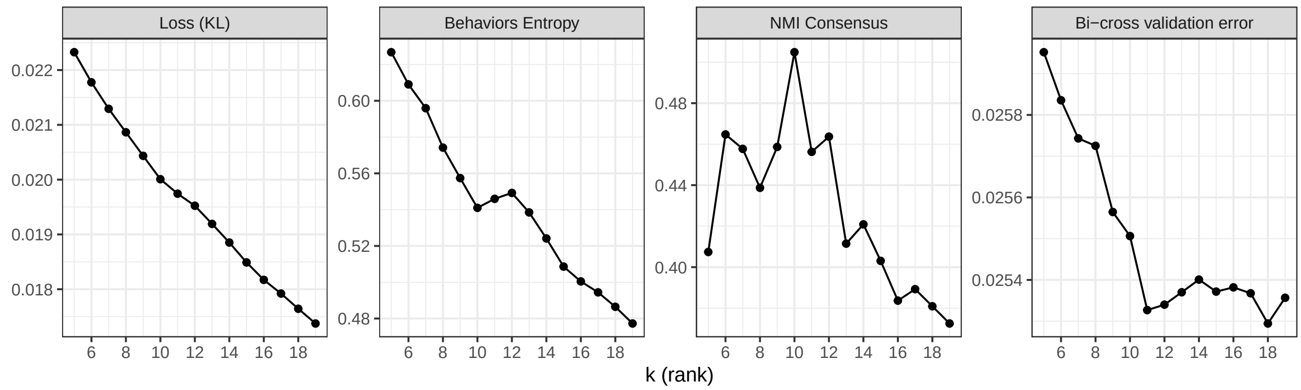

Figure S1: Rank selection metrics. Plots show the average values of the entropy for all behaviors, the bi-cross validation error, the average loss (KL divergence), and the average consensus (NMI) across 100 realizations for each factorization rank k.

7 Robustness tests

7.1 Duration of visits

In this section, we check the robustness of our results towards some of the parameters used to define the mobility of individuals. In the main paper, we considered only visits that lasted less than 4 hours. That is because some of our users are not visiting a venue but rather working there. With this upper threshold, we discarded only 5.49% of the visits to different places. Here we check that our results do not depend on the upper threshold. Using all the stays we build the matrix $X_{dur<\infty}$ and factorize it into $W_{dur<\infty} \cdot B$. We compare it with the matrix $X_{dur<4hours}$ together with the factorization $W_{dur<4hours} \cdot B$, which are the ones used in the main paper. Figure S4 shows our main results in the case in which we compute the mobility patterns using all visits, independently of their duration. As we can see, the Root Mean Square Error (RMSE) $\sqrt{\frac{1}{k}\sum_{j=1}^{k}(w_{ij,dur<\infty} - w_{ij,dur<4hours})^2}$ for each user, is very low. Furthermore, our results for the average weight by latent behavior per city and the correlation between the new weights and demographic variables and some of the urban problems are almost identical to the ones presented in the main paper. These results indicate that our main findings are largely independent of and robust to the details of how we define the upper threshold for our visits.

7.2 Sample of users

In the main text, we selected 10k users in each city to prevent an over-representation of the larger cities in the factorization. That 10k users correspond to 27% of the total number of users in Boston, 6% in Chicago, 7% in Dallas, 14% in Detroit, 6% in Los Angeles, 8.5% in Miami, 6.5% in New York, 15% in Philadelphia, 20% in San Francisco, 23% in Seattle, and 11% in Washington DC. As we can see that, our 10k user sampling overcompensates for the size of the city. To test how robust our results are to that sampling, we have also considered getting a random selection of users proportionally to the total number of users per area. In this case, we have considered that we get always 7% of the users in each area. Results for the main result of the paper with this new sampling are presented in Figure S5. In this case, we generated a new matrix factorization. Although the latent behaviors are a little bit different from the ones used in the main text, we can clearly see in Figure S5 are very similar quantitatively and qualitatively to the ones reported in the main paper. This indicates that our main findings are largely independent of and robust to the details of how we define the sample of users.

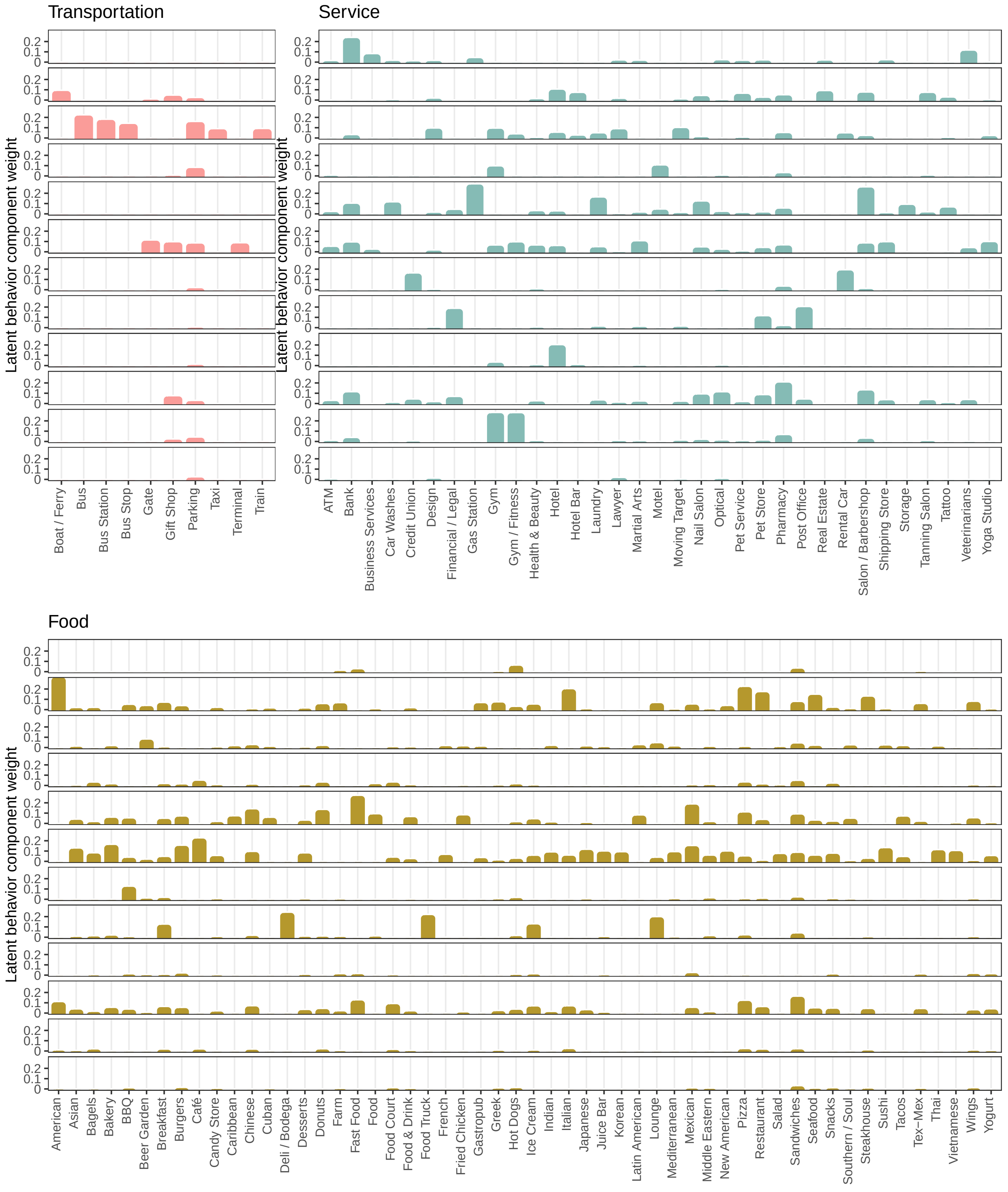

Figure S2: Latent Behavior components: Panels show all the components for the $k = 12$ latent behaviors in the different categories for 3 different type of places: Transportation, Service and Food.

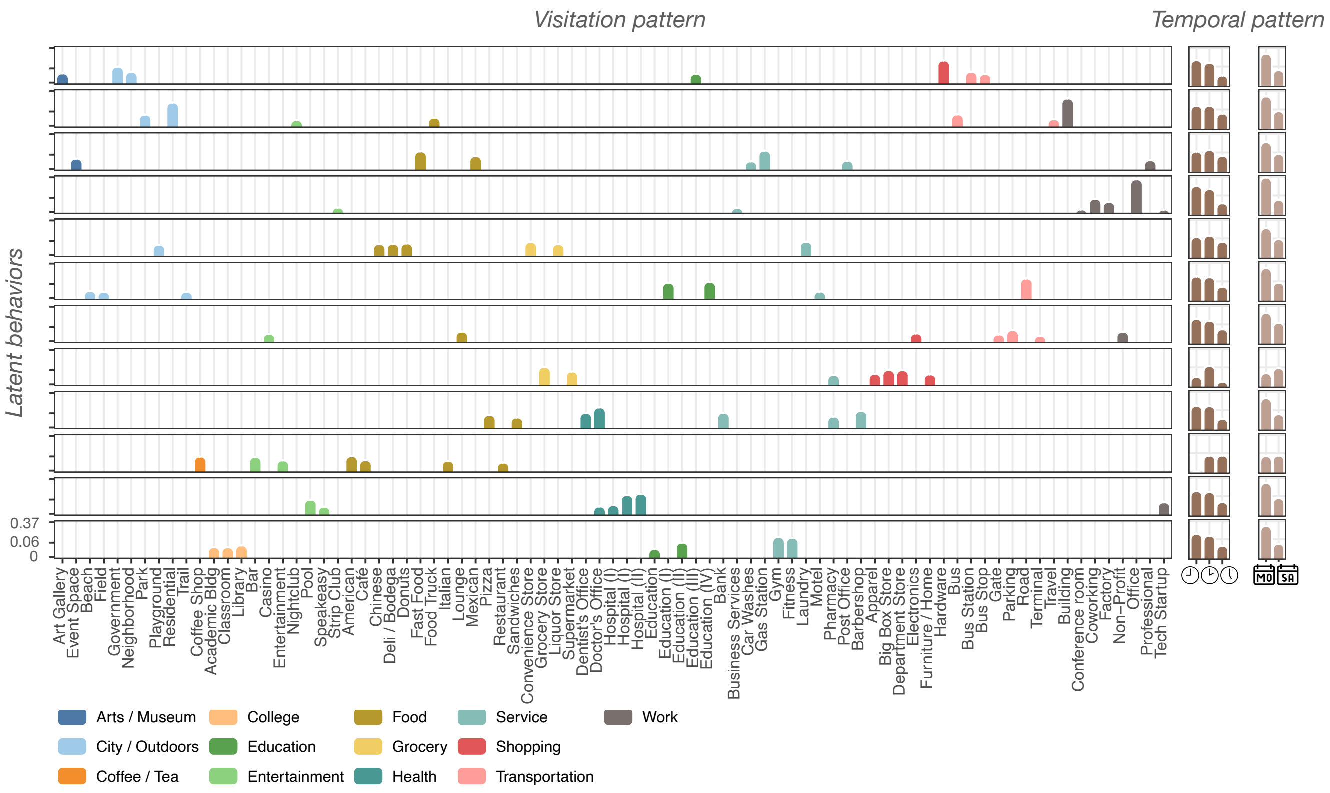

Figure S3: Latent Behavior components using LDA: Activity and temporal components for each of the $k = 12$ latent behaviors detected using LDA. For simplicity, only the top 7 categories by latent behavior are shown. Colors correspond to the different classifications of the venues. Temporal patterns correspond to the fraction of morning, afternoon, and night visits together with the fraction of weekday and weekend visits.

Table S1: Regression table for latent behavior weights as a function of different demographic variables

	Latent behavior weight w_{ij}:											
	1	2	3	4	5	6	7	8	9	10	11	12
Density	-0.077^{***}	-0.002	0.105^{***}	0.026^{***}	-0.011^{***}	0.060^{***}	-0.006^{***}	0.015^{***}	0.003^{**}	-0.078^{***}	-0.012^{***}	0.031^{***}
	(0.001)	(0.001)	(0.001)	(0.001)	(0.001)	(0.001)	(0.001)	(0.001)	(0.001)	(0.001)	(0.001)	(0.001)
Median household income	-0.015^{***}	0.029^{***}	0.006^{***}	0.018^{***}	-0.132^{***}	0.115^{***}	-0.016^{***}	-0.012^{***}	0.039^{***}	0.025^{***}	0.045^{***}	0.034^{***}
	(0.001)	(0.001)	(0.001)	(0.001)	(0.001)	(0.001)	(0.001)	(0.001)	(0.001)	(0.001)	(0.001)	(0.001)
Fraction of users of public transp.	-0.048^{***}	-0.002	0.245^{***}	0.038^{***}	-0.029^{***}	0.067^{***}	0.020^{***}	0.052^{***}	-0.017^{***}	-0.046^{***}	-0.0004	-0.036^{***}
	(0.001)	(0.001)	(0.001)	(0.001)	(0.001)	(0.001)	(0.001)	(0.001)	(0.001)	(0.001)	(0.001)	(0.001)
Fraction of black population	-0.006^{***}	-0.054^{***}	-0.006^{***}	-0.018^{***}	0.087^{***}	-0.097^{***}	0.014^{***}	-0.002^{*}	0.007^{***}	0.018^{***}	-0.001	0.014^{***}
	(0.001)	(0.001)	(0.001)	(0.001)	(0.001)	(0.001)	(0.001)	(0.001)	(0.001)	(0.001)	(0.001)	(0.001)
Constant	0.00001	0.00000	−0.00000	0.00000	0.00002	0.00001	0.00001	0.00001	0.00001	−0.00000	−0.0001	0.00001
	(0.001)	(0.001)	(0.001)	(0.001)	(0.001)	(0.001)	(0.001)	(0.001)	(0.001)	(0.001)	(0.001)	(0.001)
Observations	845,773	845,773	845,773	845,773	845,773	845,773	845,773	845,773	845,773	845,773	845,773	845,773
R^2	0.013	0.005	0.101	0.003	0.031	0.035	0.001	0.004	0.002	0.013	0.002	0.002
Adjusted R^2	0.013	0.005	0.101	0.003	0.031	0.035	0.001	0.004	0.002	0.013	0.002	0.002
Residual Std. Error	0.994	0.998	0.948	0.998	0.984	0.982	1.000	0.998	0.999	0.993	0.999	0.999

Note: $^{*}p<0.1$; $^{**}p<0.05$; $^{***}p<0.01$

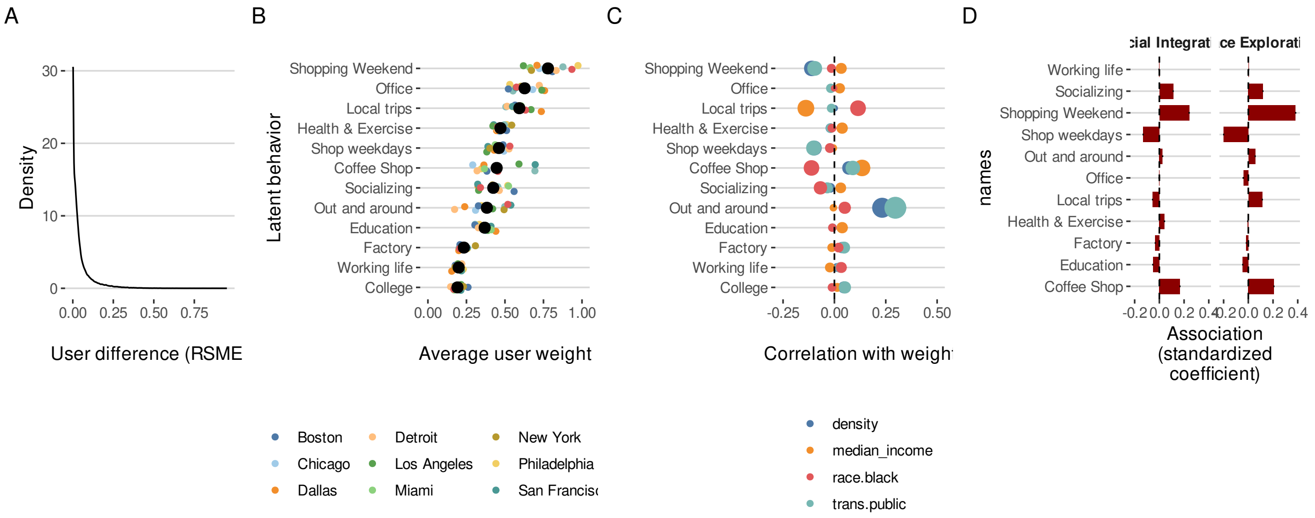

Figure S4: Robustness of our results to visit duration A) Shows the Root Mean Square Error (RMSE) between the coefficients of a user calculated with all visits and only those that lasted less than 4 hours. B) Results for the average weight for the different latent behaviors in all areas (black) and different cities considering all visits. Compare this to Figure 3 in the main paper. C) Correlation between the weight of the latent behaviors (calculated using all visits) and different demographic and urban characteristics. Compare this to Figure 3 in the main paper. D) Coefficients for the regression of Social Integration and Place Exploration with each latent behavior weight. Compare this to Figure 3 in the main paper.

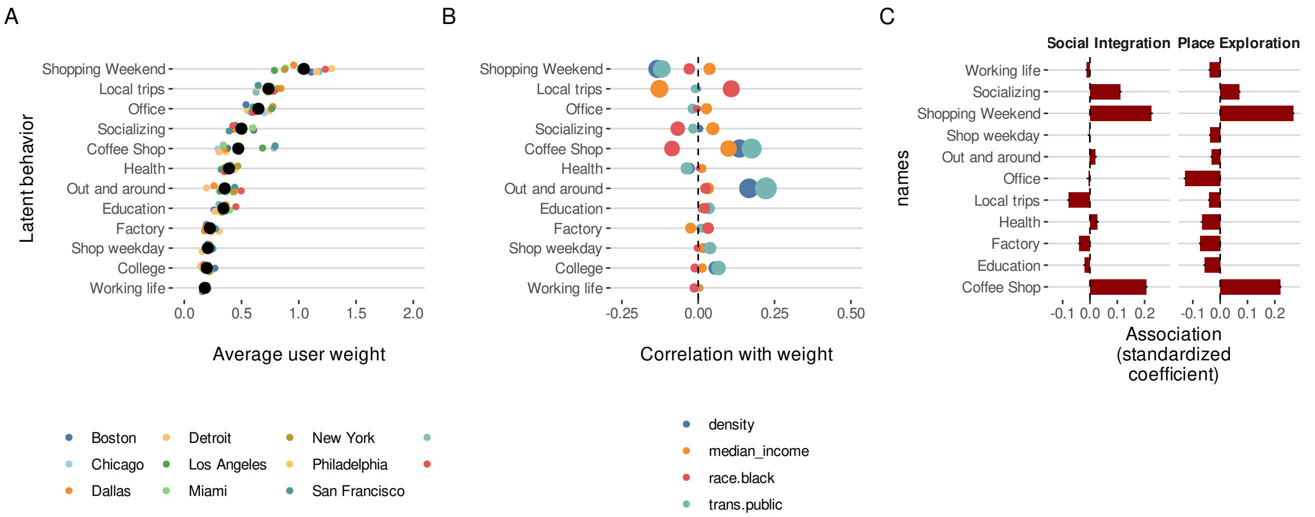

Figure S5: Robustness of our results to the sampling of users This figure is done using a sample of users proportional to the population in each city. A) Results for the average weight for the different latent behaviors in all areas (black) and different cities. Compare this to Figure 3 in the main paper. B) Correlation between the weight of the latent behaviors and different demographic and urban characteristics. Compare this to Figure 3 in the main paper. D) Coefficients for the regression of Social Integration and Place Exploration with each latent behavior weight. Compare this to Figure 3 in the main paper.

Table S2: Regression results for the models for different social, transportation, and mobility variables as a function of the weights of the latent behaviors and several demographic variables. See Supplementary Eqs. (7) and (8) . Models for Integration and Exploration are done at individual level. The rest of the models are done aggregating the variables over census tracts. Because of the normalization of weights in latent behaviors, we chose to exclude "College"(Latent Behavior 4) in models for Integration and Exploration to prevent co-linearity.

	Dependent variable X_i or X_α:					
	Integration	Exploration	Long Commute	Distance travelled	Physical Activity	No obesity
Latent Behavior 1	0.141***	−0.208***	0.125***	0.146***	−0.008	0.013
	(0.002)	(0.002)	(0.014)	(0.008)	(0.009)	(0.009)
Latent Behavior 2	−0.102***	0.122***	−0.036***	−0.038***	−0.096***	−0.013*
	(0.001)	(0.001)	(0.012)	(0.006)	(0.008)	(0.007)
Latent Behavior 3	−0.020***	0.052***	0.247***	−0.186***	−0.131***	−0.105***
	(0.002)	(0.001)	(0.017)	(0.010)	(0.012)	(0.011)
Latent Behavior 4			0.006	−0.014**	−0.078***	−0.079***
			(0.011)	(0.006)	(0.007)	(0.007)
Latent Behavior 5	0.071***	0.119***	0.121***	0.051***	0.223***	0.163***
	(0.002)	(0.002)	(0.014)	(0.007)	(0.009)	(0.009)
Latent Behavior 6	−0.155***	0.214***	−0.076***	−0.118***	−0.086***	−0.121***
	(0.001)	(0.001)	(0.014)	(0.007)	(0.009)	(0.009)
Latent Behavior 7	0.014***	0.001	0.053***	−0.030***	−0.011*	−0.001
	(0.001)	(0.001)	(0.010)	(0.005)	(0.006)	(0.006)
Latent Behavior 8	0.043***	−0.024***	0.095***	−0.014**	0.001	0.010
	(0.001)	(0.001)	(0.011)	(0.006)	(0.007)	(0.007)
Latent Behavior 9	0.070***	−0.052***	0.135***	−0.013**	−0.0004	0.005
	(0.002)	(0.002)	(0.010)	(0.006)	(0.007)	(0.006)
Latent Behavior 10	−0.205***	0.382***	0.127***	0.117***	−0.041***	−0.023***
	(0.002)	(0.002)	(0.012)	(0.007)	(0.008)	(0.008)
Latent Behavior 11	−0.020***	−0.016***	0.125***	−0.050***	−0.046***	−0.043***
	(0.002)	(0.002)	(0.011)	(0.006)	(0.007)	(0.007)
Latent Behavior 12	0.023***	−0.053***	0.049***	−0.051***	−0.030***	−0.035***
	(0.002)	(0.002)	(0.012)	(0.007)	(0.008)	(0.008)
Population Density	0.006***	0.019***	−0.082***	−0.028***	0.024***	0.007
	(0.001)	(0.001)	(0.016)	(0.009)	(0.008)	(0.008)
Median household income	−0.103***	0.070***	0.0002	0.690***	−0.528***	−0.307***
	(0.001)	(0.001)	(0.012)	(0.007)	(0.008)	(0.008)
Fraction of users of public transp.	0.054***	0.054***	0.158***	−0.069***	0.029**	−0.037***
	(0.001)	(0.001)	(0.016)	(0.011)	(0.013)	(0.012)
Fraction of black population	0.090***	0.001	0.081***	−0.093***	0.062***	0.377***
	(0.001)	(0.001)	(0.010)	(0.006)	(0.007)	(0.007)
Constant	0.171***	−0.161***	−0.017	−0.145***	−0.151***	−0.187***
	(0.006)	(0.006)	(0.040)	(0.019)	(0.027)	(0.026)
Observations	845,773	845,773	7,023	13,179	5,710	5,710
R^2	0.164	0.260	0.508	0.724	0.820	0.837
Adjusted R^2	0.164	0.260	0.506	0.724	0.819	0.836
Residual Std. Error	0.914	0.861	0.703	0.526	0.425	0.405
	(df = 845747)	(df = 845747)	(df = 6998)	(df = 13154)	(df = 5685)	(df = 5685)
Note:						*p<0.1; **p<0.05; ***p<0.01

Table S3: Regression results for the models for different social, transportation, and mobility variables as a function only of demographic variables. See Supplementary Eqs. (7) and (8), and compare with Table S2.

	Dependent variable X_i or X_α:					
	Integration	Exploration	Long commute	Distance travelled	Physical Activity	No obesity
Population Density	−0.0004	0.021***	−0.097***	−0.118***	−0.031***	−0.048***
	(0.001)	(0.001)	(0.016)	(0.011)	(0.010)	(0.009)
Median household income	−0.135***	0.076***	−0.072***	0.565***	−0.728***	−0.474***
	(0.001)	(0.001)	(0.010)	(0.007)	(0.008)	(0.007)
Fraction of users of public transp.	0.048***	0.049***	0.105***	−0.372***	−0.136***	−0.185***
	(0.002)	(0.002)	(0.016)	(0.010)	(0.013)	(0.012)
Fraction of black population	0.108***	−0.005***	0.154***	−0.057***	0.174***	0.461***
	(0.001)	(0.001)	(0.010)	(0.006)	(0.008)	(0.007)
Constant	0.140***	−0.120***	−0.161***	−0.086***	−0.250***	−0.264***
	(0.006)	(0.006)	(0.040)	(0.021)	(0.031)	(0.029)
Observations	845,773	845,773	7,023	13,179	5,710	5,710
R^2	0.059	0.025	0.454	0.625	0.739	0.781
Adjusted R^2	0.059	0.025	0.453	0.625	0.739	0.781
Residual Std. Error	0.970	0.987	0.740	0.613	0.511	0.468
	(df = 845758)	(df = 845758)	(df = 7010)	(df = 13166)	(df = 5697)	(df = 5697)
Note:						*p<0.1; **p<0.05; ***p<0.01

References

[1] Hariharan, R. & Toyama, K. Project lachesis: parsing and modeling location histories. In *International Conference on Geographic Information Science*, 106–124 (Springer, 2004).

[2] Foursquare Venue Category Hierarchy. `https://developer.foursquare.com/docs/build-with-foursquare/categories/` (2020). Accessed: 09-12-2020.

[3] Aslak, U. & Alessandretti, L. Infostop: scalable stop-location detection in multi-user mobility data. *arXiv preprint arXiv:2003.14370* (2020).

[4] United States Census Bureau. Core-Based Statistical Areas. `https://www.census.gov/topics/housing/housing-patterns/about/core-based-statistical-areas.html` (2000). Accessed: 22-06-2019.

[5] Moro, E., Calacci, D., Dong, X. & Pentland, A. Mobility patterns are associated with experienced income segregation in large US cities. *Nature Communications* **12**, 4633 (2021).

[6] Lin, X. & Boutros, P. C. Optimization and expansion of non-negative matrix factorization. *BMC Bioinformatics* **21**, 7 (2020).

[7] Lin, X. & Paul C Boutros. *NNLM: Fast and Versatile Non-Negative Matrix Factorization* (2020). URL `https://github.com/linxihui/NNLM`. R package version 0.4.4.

[8] Graells-Garrido, E., Caro, D. & Parra, D. Toward Finding Latent Cities with Non-Negative Matrix Factorization. *arXiv* (2018). `1801.09093`.

[9] Møllgaard, P. E., Lehmann, S. & Alessandretti, L. Understanding components of mobility during the COVID-19 pandemic. *Philosophical Transactions of the Royal Society A* **380**, 20210118 (2022).

Table S4: Regression results for the models for different social, transportation, and mobility variables as a function only of behavioral latent weights. See Supplementary Eqs. (7) and (8), and compare with Table S2.

	Dependent variable X_i or X_α:					
	Integration	Exploration	Long commute	Distance travelled	Physical Activity	No obesity
Latent Behavior 1	0.136***	−0.214***	0.119***	0.236***	−0.059***	−0.030**
	(0.002)	(0.002)	(0.014)	(0.011)	(0.013)	(0.013)
Latent Behavior 2	−0.113***	0.122***	−0.045***	0.056***	−0.183***	−0.106***
	(0.002)	(0.001)	(0.012)	(0.008)	(0.010)	(0.010)
Latent Behavior 3	0.003*	0.059***	0.293***	−0.381***	−0.045***	0.001
	(0.002)	(0.001)	(0.017)	(0.011)	(0.014)	(0.014)
Latent Behavior 4			0.006	0.020**	−0.101***	−0.079***
			(0.011)	(0.008)	(0.009)	(0.009)
Latent Behavior 5	0.094***	0.109***	0.139***	−0.182***	0.405***	0.381***
	(0.002)	(0.002)	(0.013)	(0.009)	(0.011)	(0.011)
Latent Behavior 6	−0.167***	0.223***	−0.084***	0.126***	−0.273***	−0.320***
	(0.001)	(0.001)	(0.013)	(0.009)	(0.011)	(0.011)
Latent Behavior 7	0.018***	−0.001	0.060***	−0.072***	0.020**	0.048***
	(0.001)	(0.001)	(0.010)	(0.007)	(0.008)	(0.008)
Latent Behavior 8	0.047***	−0.026***	0.095***	−0.015**	−0.013	0.001
	(0.001)	(0.001)	(0.011)	(0.008)	(0.009)	(0.009)
Latent Behavior 9	0.066***	−0.053***	0.138***	0.077***	−0.048***	0.002
	(0.002)	(0.002)	(0.010)	(0.008)	(0.009)	(0.009)
Latent Behavior 10	−0.211***	0.376***	0.129***	0.180***	−0.091***	−0.032***
	(0.002)	(0.002)	(0.012)	(0.009)	(0.011)	(0.011)
Latent Behavior 11	−0.025***	−0.018***	0.122***	0.031***	−0.101***	−0.070***
	(0.002)	(0.002)	(0.011)	(0.008)	(0.010)	(0.010)
Latent Behavior 12	0.019***	−0.053***	0.060***	0.040***	−0.101***	−0.037***
	(0.002)	(0.002)	(0.012)	(0.009)	(0.011)	(0.011)
Constant	0.129***	−0.136***	0.012	−0.032	−0.233***	−0.255***
	(0.006)	(0.006)	(0.041)	(0.025)	(0.037)	(0.037)
Observations	845,799	845,799	7,024	13,180	5,711	5,711
R^2	0.135	0.254	0.493	0.499	0.669	0.674
Adjusted R^2	0.135	0.254	0.491	0.498	0.668	0.673
Residual Std. Error	0.930	0.864	0.713	0.709	0.576	0.572
	(df = 845777)	(df = 845777)	(df = 7003)	(df = 13159)	(df = 5690)	(df = 5690)
Note:						*p<0.1; **p<0.05; ***p<0.01

[10] Aledavood, T., Kivimäki, I., Lehmann, S. & Saramäki, J. Quantifying daily rhythms with non-negative matrix factorization applied to mobile phone data. *Scientific Reports* **12**, 5544 (2022).

[11] Hutchins, L. N., Murphy, S. M., Singh, P. & Graber, J. H. Position-dependent motif characterization using non-negative matrix factorization. *Bioinformatics* **24**, 2684–2690 (2008).

[12] Brunet, J.-P., Tamayo, P., Golub, T. R. & Mesirov, J. P. Metagenes and molecular pattern discovery using matrix factorization. *Proceedings of the National Academy of Sciences* **101**, 4164–4169 (2004).

[13] Owen, A. B. & Perry, P. O. Bi-cross-validation of the SVD and the nonnegative matrix factorization. *The Annals of Applied Statistics* **3**, 564–594 (2009). `0908.2062`.

[14] Hoyer, P. O. Non-negative Matrix Factorization with Sparseness Constraints. *Journal of Machine Learning Research* 1457–1569 (2004).

[15] Danon, L., Diaz-Guilera, A., Duch, J. & Arenas, A. Comparing community structure identification. *Journal Of Statistical Mechanics-Theory And Experiment* P09008 (2005). URL `http://iopscience.iop.org/1742-5468/2005/09/P09008/?ejredirect=iopscience`.

[16] United States Census Bureau. 2013-2017 American Community Survey 5-year Estimates. `https://www.census.gov/programs-surveys/acs` (2019). Accessed: 2020-12-04.
